# Positive pressure in bamboo is generated in stems and rhizomes, not in roots

**DOI:** 10.1093/aobpla/plae040

**Published:** 2024-07-19

**Authors:** Joseph M Michaud, Kerri Mocko, H Jochen Schenk

**Affiliations:** Department of Biological Science, California State University Fullerton, 800 N. State College Blvd., Fullerton, CA 92831, USA; Department of Biological Science, California State University Fullerton, 800 N. State College Blvd., Fullerton, CA 92831, USA; Department of Biological Science, California State University Fullerton, 800 N. State College Blvd., Fullerton, CA 92831, USA

**Keywords:** bamboo, *Bambusa oldhamii*, guttation, plant ecophysiology, plant hydraulics, plant water relations, positive pressure, root pressure, sap flow, stem pressure, xylem

## Abstract

Bamboos stand out among other tall plants in being able to generate positive pressure in the xylem at night, pushing water up to the leaves and causing drops to fall from leaf tips as guttation that can amount to a steady nocturnal ‘bamboo rain’. The location and mechanism of nocturnal pressure generation in bamboos are unknown, as are the benefits for the plants. We conducted a study on the tall tropical bamboo species *Bambusa oldhamii* (giant timber bamboo) growing outdoors in southern California under full irrigation to determine where in the plant the nocturnal pressure is generated, when it rises in the evening, and when it dissipates in the morning. We hypothesized that the build-up of positive pressure would be triggered by the cessation of transpiration-driven sap flow and that resumption of sap flow in the morning would cause the pressure to dissipate. Nocturnal pressure was observed in mature stems and rhizomes, but never in roots. The pressure was episodic and associated with stem swelling and was usually, but not always, higher in rhizomes and basal stems than in stems at greater height. Time series analyses revealed that dry atmospheric conditions were followed by lower nocturnal pressure and rainfall events by higher stem pressure. Nocturnal pressure was unrelated to sap flow and even was generated for a short time in isolated stem pieces placed in water. We conclude that nocturnal pressure in bamboo is not ‘root pressure’ but is generated in the pseudo-woody rhizomes and stems. It is unrelated to the presence or absence of sap flow and therefore must be created outside of vessels, such as in phloem, parenchyma, or fibres. It is unlikely to be a drought adaptation and may benefit the plants by maximizing stem water storage for daytime transpiration or by transporting nutrients to the leaves.

## Teaser

Bamboos stand out among other tall plants in being able to push up water under positive pressure at night all the way to the leaves, causing drops to fall from leaf tips as guttation that can amount to a steady nocturnal ‘bamboo rain’. In research on the tall tropical bamboo species *Bambusa oldhamii* (giant timber bamboo) growing outdoors in southern California under full irrigation, we found that nocturnal pressure occurred in mature stems and rhizomes, but never in roots. Dry and hot atmospheric conditions were followed by lower nocturnal pressure and cool temperatures and rainfall by higher pressure. Nocturnal pressure was unrelated to sap flow and even was generated for a short time in isolated stem pieces placed in water. Positive pressure at night may benefit the plants by maximizing stem water storage for daytime transpiration or by transporting nutrients to the leaves.

## Introduction

Vascular plants have two ways of moving water through xylem conduits: transpiration-driven transport via the cohesion-tension mechanism (Askenasy 1895; Dixon and Joly 1895), under negative pressure and under positive pressure in the absence of transpiration (Kramer and Boyer 1995; Singh 2016*b*; Schenk *et al.* 2021). Quantitatively, transpiration-driven transport is the more dominant mechanism by far, as water moves as vapour out of leaves into the atmosphere. Water transport under positive pressure only refills plant tissues and in some cases pushes small amounts of water out as guttation through hydathodes located mostly on leaves.

Perhaps because of the smaller quantities of water involved, the positive pressure mode has been the subject of much less research and is only briefly mentioned in textbooks where it is traditionally referred to as ‘root pressure’, even though it can manifest in roots, stems, rhizomes and leaves (Kramer and Boyer 1995; Schenk *et al.* 2021). Regardless of where it is generated, the pressure that is measured in stems occurs in non-living xylem conduits (i.e. the apoplast) of most herbaceous plants, pseudo-woody monocots, such as palms and bamboos, vines, lianas, ferns and in many trees except conifers (Schenk *et al.* 2021). Based on recent reviews (Singh 2016*b*; Schenk *et al.* 2021), it appears that most angiosperms can produce positive pressure in the xylem apoplast. Positive pressure can develop temporarily in a leafless stage, for example before leaf-out in spring or as nocturnal positive pressure that alternates with negative pressure during the day when the plants are transpiring (Schenk *et al.* 2021).

Nocturnal positive pressure in plants is a particularly puzzling phenomenon, as the mechanisms behind its creation and potential benefits for the plant are still largely unknown. The extensive literature on this subject has been summarized in recent reviews (Singh 2016*b*; Schenk *et al.* 2021). Nocturnal pressure is usually observed as guttation from leaves or is measured in cut stems and for most plants it is not known if it is generated in the roots or potentially in rhizomes, stems, or even leaves. Hypothesized functions of nocturnal positive pressure include rehydration of tissues and refilling of embolized conduits (Singh 2016*b*; Schenk *et al.* 2021), as well as transport of nutrients and hormonal signals to leaves (Feild and Arens 2007), but the adaptative benefits of nocturnal pressure for plant fitness are essentially unknown.

Bamboos are a large and important group of grasses in which nocturnal positive pressure appears to play an important role in water relations, especially for stem tissue rehydration overnight (Saha *et al.* 2009; Wang *et al.* 2011; Yang *et al.* 2012, 2015). Bamboos are fast-growing pseudo-woody plants that cover vast areas of tropical to temperate latitudes worldwide (Du *et al.* 2018), including more than 1400 species (Kelchner 2013). Many are used commercially as sustainable building materials, a source of fibre, food and bioenergy crops with very high carbon-accumulation rates. Bamboos are the tallest vascular plants known to generate positive pressure at night (Wang *et al.* 2011; Cao *et al.* 2012; Schenk *et al.* 2021), pushing water up to the leaves even in the largest species, causing drops of water to fall from leaf tips (guttation) that can amount to a steady nocturnal ‘bamboo rain’ (Molisch 1898).

Positive nocturnal pressure in bamboo stems universally has been referred to as ‘root pressure’ (e.g. Cochard *et al.* 1994; Marine 2009; Wang *et al.* 2011; Cao *et al.* 2012; Yang *et al.* 2012, 2024; Mei *et al.* 2016; Ocheltree *et al.* 2020; Dai *et al.* 2023), even though the origin of the pressure in bamboos in roots vs. rhizomes or stems has never been determined in any of these studies. The purpose of our study was to determine where in the plant the nocturnal pressure is generated and to collect information that will help to determine how that pressure is created in the evening, and how it dissipates in the morning. We hypothesized that the build-up of positive pressure in the evening would be triggered by the cessation of transpiration-driven sap flow, and that resumption of sap flow in the morning would cause the pressure to dissipate. Alternatively, pressure build-up and dissipation could be independent of transpiration and sap flow.

How does positive nocturnal pressure benefit bamboos? Previous research on several bamboo species has found that nocturnal pressure increases hydraulic conductance in stems and leaves overnight, increasing stem water rehydration and storage (Saha *et al.* 2009; Yang *et al.* 2012, 2015). Accordingly, we hypothesized that stem pressure would result in increasing stem diameter, which is an indirect measure of stem water storage (Yang *et al.* 2015). We also tested the hypothesis that the magnitude of nocturnal pressure would be positively correlated with the vapour pressure deficit (VPD) of the air during midday and negatively correlated with VPD during the night. This hypothesis is based on the observation that high VPD during the day is associated with high stomatal conductance (Buckley 2005, 2019) and transpiration rates that would deplete water storage in stems and that low VPD during the night tends to be associated with leaf guttation (Frey-Wyssling 1941; Singh 2016*a*).

Based on previous findings, we chose a tall bamboo species, the giant timber bamboo *Bambusa oldhamii* Munro, as our study system, because taller bamboos tend to generate the highest pressures (Cao *et al.* 2012). The species is a large, clump-forming bamboo that is native to tropical and subtropical regions in China and Taiwan. It has been widely planted outside of its natural range for a variety of uses as building material, as a fibre source, and as food.

## Material and Methods

### Study system

*Bambusa oldhamii* Munro plants were studied continuously between July 2020 and July 2021 on the campus of California State University, Fullerton in Fullerton, California, USA. The plants are part of irrigated campus landscaping and form a dense stand (average density 9 stems m^−2^) in a bed of 17.5 m length and 2.5 m width, with concrete barriers on all sides. It is unknown if the shoots are all connected by functional rhizomes and if they are composed of one or more genets. Mature non-growing shoots selected for the study on average measured approximately 6.5 cm in diameter (without taper for most of the stem length) and 12–18 m in height, as determined by cutting of a subset of stems. Watering of the stand was controlled via a drip line system, set up to water the plants daily at 21:30. Based on monitoring of daily maximum sap flow rates (see methods below and **Supporting Information**),the plants did not experience soil water limitations throughout the experiment.

Because the occurrence of nocturnal positive pressure was found to occur in episodes that lasted for several days to a few weeks under daily irrigation, we examined the relationship between these episodes and past and current weather. Weather conditions of the study site, including daily maxima and minima ambient temperature, daily precipitation, atmospheric pressure, relative humidity and wind speed were taken from the Fullerton Municipal Airport weather station (National Weather Service, Network ID GHCND:USW00003166) weather station, approximately 8 km from the study site. The City of Fullerton is located on mostly flat terrain, and therefore has little local variation in weather. Daily minima and maxima of VPD were calculated from these data.

### Pressure measurements

To measure positive stem pressure in bamboo culms (hereafter referred to as stems), board-mounted pressure sensors (model 26PCCFJ6G, Honeywell, Charlotte, NC) were connected to loggers (model UX120-006 HOBO 4—Channel Analog Data Logger, Onset Computer Corp., Bourne, MA). Sensors were calibrated using a custom pressure apparatus with a pressure gauge (model 75514-26B55 300 PSI Digital Test Gauge, 3D Instruments, Anaheim, CA) where the needle like side of the board-mounted pressure sensor was attached to the chamber with Tygon tubing and clamp and the sensor was connected to a logger (model UX120-006 HOBO 4—Channel Analog Data Logger, Onset Computer Corp., Bourne, MA). The pressure of the chamber was manipulated from 0.7 to 96.5 kPa (in 5–7 increments) and the reported voltages of the pressure sensors were recorded, from which a linear calibration slope-intercept formula was calculated. A subset of sensors was also calibrated up to 450 kPa using a pressure chamber (model 1505D Pressure Chamber Instrument, PMS Instrument Company, Albany, OR), where the sensors continued to show a linear relationship to pressure changes. Every pressure sensor was individually calibrated.

To install on bamboo stems, the sensor’s needle-like plastic connector was inserted tightly into a pre-drilled 1.5 cm deep, 1.59 mm diameter hole. Care was taken to ensure that the holes did not penetrate the hollow centre of the stems. No sealant was required to create tight connections. To determine if positive pressure is produced from the rhizome, at the base of the stem, or different stem heights, pressure sensors were installed at various stem heights from the base of the stem to the lower canopy, and on the rhizome, which were located very close to the soil surface. The installation heights differed between experiments and are listed in the figures. Measurements of stem pressure were logged every minute using data loggers (model UX120-006 HOBO 4—Channel Analog Data Logger, Onset Computer Corp., Bourne, MA).

Root pressure of *B. oldhamii* was measured at 1-minute intervals using the same board mount pressure sensors and loggers used for stem pressure, with 3 cm length clear water-filled Tygon tubing tightly clamped to the sensor and over the end of a freshly cut root. After sensor installation, the setup was covered with the same soil that had been removed to access the root Roots of mature bamboo plants are all adventitious and lack secondary growth. They measured approximately 3 mm in diameter and were at less than 10 cm depth, with sensors being installed on both sides of the cut roots.

### Measurement of stem sap flux and stem diameter variations

To determine how sap flow coincides with the cycles of positive and negative pressure in bamboo stems, sap flow metres (model SFM1 Sap Flow Meter, ICT International, Armidale, NSW, Australia) were used to measure high, low and reverse water flow rates in the stems of bamboo. Measurements of sap flux density were automatically calculated (cm/hr) using the Heat Ratio Method (Burgess *et al.* 2001), with measurements automatically taken every 10 min. To measure diurnal changes in stem diameter as an indirect measure of water storage in bamboo stems, point dendrometers (model Ecomatik DD-L, Ecomatik, Dachau, Germany) were installed to track changes in diameter (μm) of stems and were connected to loggers (model UX120-006 HOBO 4—Channel Analog Data Logger, Onset Computer Corp., Bourne, MA, USA), with measurements taken automatically every minute. We also attempted to measure stem water potential with a stem psychrometer (model PSY1, ICT International, Armidale, NSW, Australia). Unfortunately, positive stem pressure caused nightly refilling of the psychrometer chamber with liquid water, making measurements largely impossible, except for a brief period in July 2020 **[see**  **Supporting information—Fig. S2****]**.

### Cutting experiments

Experiment 1—To determine if the origin of positive pressure was in roots, rhizomes, or stems, a cutting experiment was performed to determine if cut stems without roots or rhizomes would continue to produce positive pressure in the presence of a water source. Pressure sensors were installed on four mature stems at the top of the first (29.25 ± 10.5 cm above ground) and fourth internodes (128.25 ± 12.6 cm above ground) on 18 May 2022. Nightly positive stem pressure was observed from all eight pressure sensors, ranging from 100 to 350 kPa over the following week. Two stem segments were cut from each stem at 9:30 in the morning on 26 May 2022, using a cleaned handsaw, one encompassing the lower part of the first to the lower part of the fourth internode and one the lower part of the fourth to the lower part of the eight internode. The cut stems were immediately transported to the lab and four randomly selected stem pieces were placed in a container with water submerging 20 cm of the cut stem and the other four without water. Measurements of stem pressure on each segment resumed at 11:00 in the morning on 26 May and continued for the following week. Mean pressure measurements after cutting that had 95% confidence intervals above 0 kPa were taken as rejecting the null hypothesis that no positive pressure would be produced after cutting the stems.

Experiment 2—An alternative stem-cutting experiment was performed to determine the relationship between stem pressure and sap flow. Pressure sensors, point dendrometers and sap flow sensors (see model information above) were installed on four stems to measure changes in these physiological variables in response to cutting off the tops of the plants. Pressure and dendrometer measurements were collected automatically every minute via data loggers (see model information above), with sap flow measurements logged every 10 min. After measuring pressure, diameter variations and sap flow for a week, three bamboo stems were fully cut on 25 June 2021 at 11:00 in the morning at approximately 2.4 m height above ground to remove the majority of the stem and all of the canopy. One stem was left uncut. Measurements of physiological variables continued for 12 days after cutting. The null hypothesis to be tested in this experiment was that build-up and dissipation of positive pressure would be unrelated to the cessation and beginning of sap flow. This hypothesis was tested by registering periods during which positive nocturnal pressure was measured in all three cut stems, none of which showed significant sap flow after cutting.

### Data analysis

The magnitude of nocturnal positive pressure measured in bamboo varied greatly over time and between sensor locations on the plants. To achieve the main purpose of our study, which was to determine where in the plant the nocturnal pressure is generated, we documented diurnal patterns of average pressure in roots vs. rhizomes vs. different stem heights and presented them in graphical form. The occurrence of positive pressure in all replicates of roots, rhizomes and stem heights at a given time was taken as evidence of positive pressure at that location without reference to the magnitude of that pressure.

The data for pressure, sap flow, stem diameter variations and weather were time series, mostly with diurnal fluctuations. To analyse relationships between these variables, we conducted cross-correlation and autocorrelation analyses using the statistical software package PAST (version 4.02; Hammer *et al.* 2001). Tests of the null hypothesis of no correlation were calculated according to Davis (1986), resulting in probabilities for each time lag, which are not corrected for multiple comparisons, so must be interpreted with caution. Cross-correlation analyses of relationships between pressure and weather data were conducted using daily summary data for a combined 293 measurement days between July 2020 and July 2021 of maximum nocturnal pressure, maximum and minimum daily VPD and temperature, daily precipitation and daily maximum wind velocity, only allowing for negative time lags up to 20 days, because pressure may be related to past but not to future weather. Analyses of relationships among pressure, sap flow and stem diameter variations were performed for two time periods, in July 2020 (11 days) and March 2021 (14 days), using 10-min intervals, allowing for both positive and negative time lags of up to 3 hr between the variables. From these cross-correlations we determined instant correlations at time lag zero, as well as positive and negative lag effects between variables by comparing the magnitude of correlation coefficients and their probabilities as a function of the time lags.

## Results

**Figure 1.**
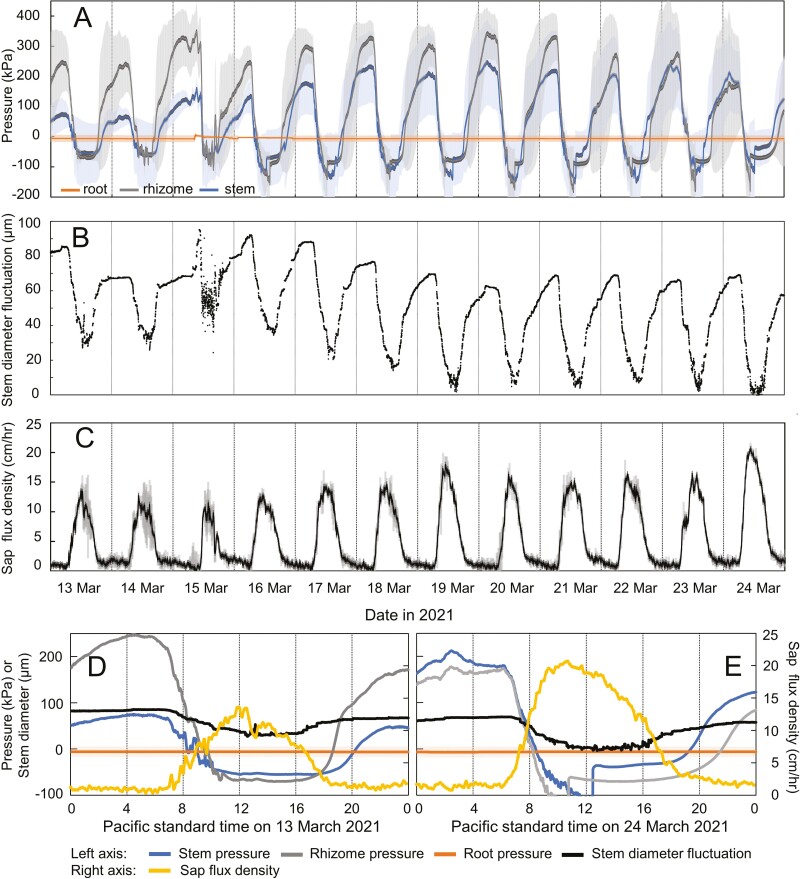
Pressure, stem diameter fluctuations, and sap flow in *B. oldhamii* measured in March 2021. Mean daily temperatures during this period varied from 11.1 to 16.4 °C and it rained on 13 March (33 mm). A. Root pressure (mean ± SE of *n* = 6), rhizome pressure (mean ± SE of *n* = 3), and stem pressure (mean ± SE of *n* = 4). B. Stem diameter fluctuations measured with a point dendrometer (*n* = 1). C. Sap flow measured using heat pulse sensors (mean ± SE of *n* = 2). D. Same data as panels A–C combined for 13 March 2021. E. Same data as panels A–C combined for 24 March 2021.

**Figure 2.**
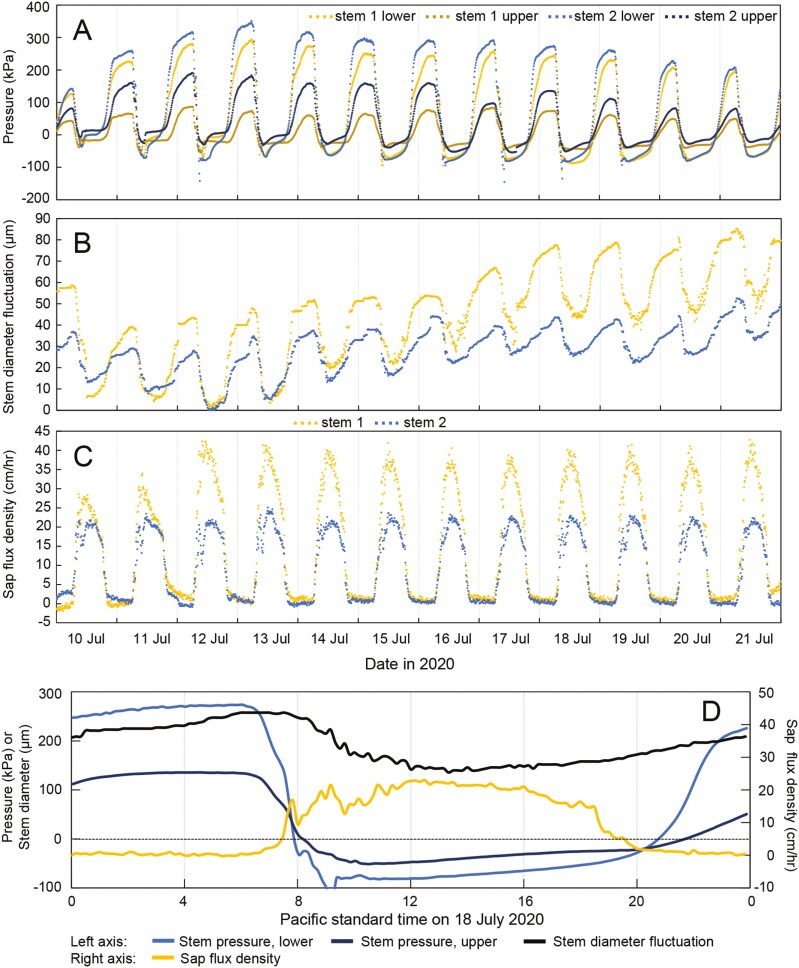
Stem pressure, stem diameter fluctuations, and sap flow in *B. oldhamii* measured in July 2020. Mean daily temperatures during this period varied from 21.4 to 26.9°C with a cooling trend over time, and there was no rain. A. Pressure measured in two stems at two different heights (10 cm and 150 cm above ground). Each curve represents the average of two pressure measurements at each height per stem. B. Stem diameter fluctuations measured with point dendrometer at two stems at 147 cm height. C. Sap flux density measured using heat pulse sensors on two stems at 135 cm height. D. Same data as in panels A–C combined for stem 2 on 18 July 2020.

**Figure 3.**
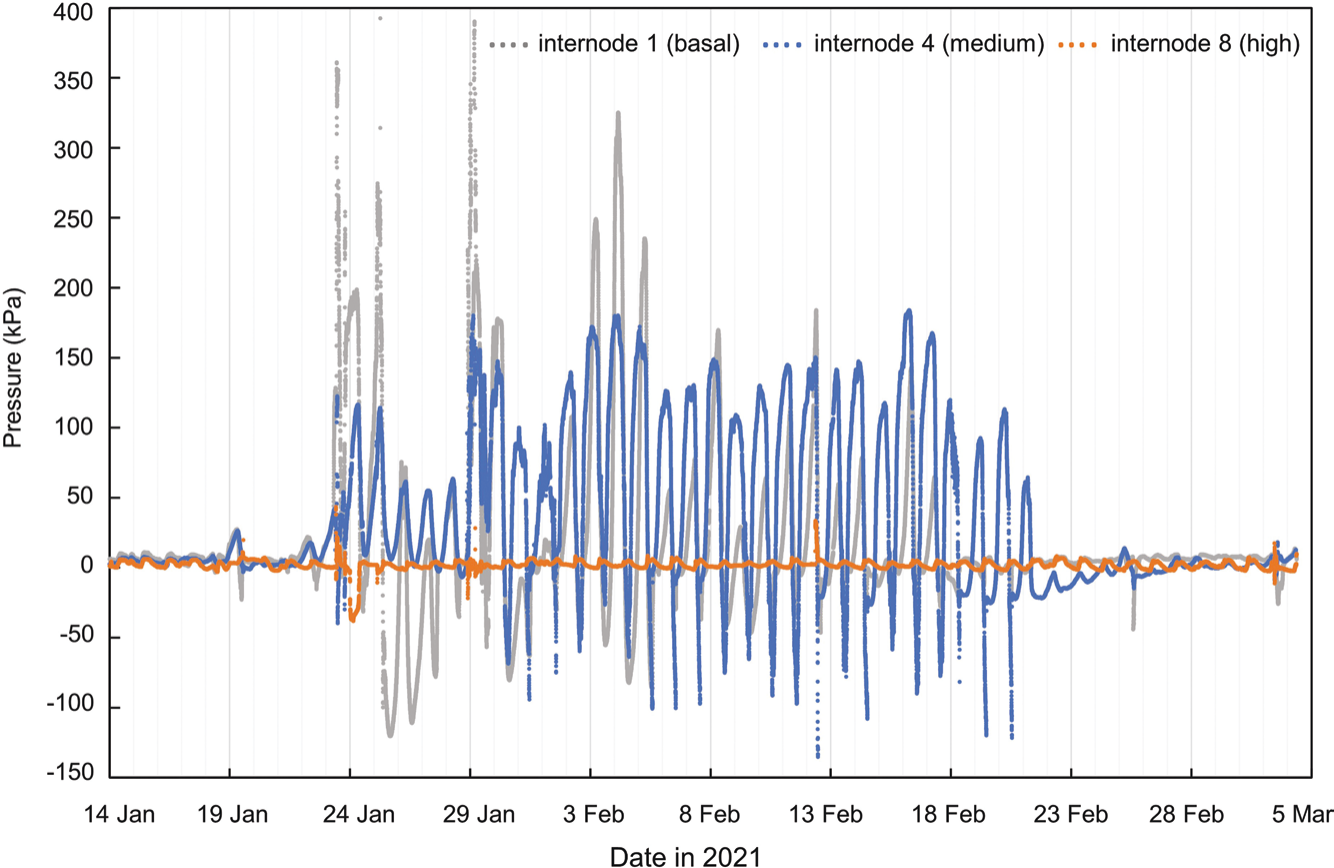
Mean pressure at different heights in *B. oldhamii* measured from January to March 2021. Mean daily temperatures during this period varied from 8.9 to 21.4 °C, with a relatively warm period ending on 22 January. The highest wind speeds were recorded on 20 February. Rainfall (394 mm) occurred from 24 to 30 January. The mean heights of pressure measurements were 27.5 cm above ground for internode 1 (basal, grey, *n* = 3), 121.5 cm for internode 4 (medium, *n* = 3), and 318.0 cm for internode 8 (high, *n* = 2).

No positive pressure and no diurnal pressure fluctuations of any kind were detected in the roots (Fig. 1a). Over several measurement periods, the magnitude of the nocturnal stem pressure usually declined with height, with rhizomes mostly showing higher pressure than stems (Fig. 1a, d) and basal internodes having higher pressure than internodes higher up (Figs. 2a and 3). There were some periods, however, when nocturnal rhizome pressure did not exceed stem pressure (Fig. 1a, e) and when higher pressures were measured at higher internodes compared to more basal ones (e.g. Fig. 3).

Diurnal patterns of stem pressure and stem diameter fluctuations coincided, with both pressure and stem diameter increasing from a minimum in the late afternoon and peaking after midnight (Figs. 1 and 2), while diurnal patterns of sap flow were the opposite, peaking during midday (Figs. 1 and 2). Stem diameter variations were positively cross-correlated with stem pressure, with stem pressure leading stem diameter slightly by 10–50 min **[see**  **Supporting Information—Fig. S1****]**. The strongest negative correlation between sap flow and stem pressure was found for a time lag of sap flow leading pressure by 50–80 min **[see**  **Supporting Information—Fig. S1****]**. Time series for all three variables were strongly autocorrelated **[see**  **Supporting Information—Fig. S1****]**, so cross-correlations between the variables must be interpreted with caution.

Periods of positive stem pressure were episodic (Fig. 3), and there were periods lasting from a few days to several weeks when no significant positive pressure was detected. The lowest stem water potential we observed was about −1 MPa in July 2020 **[see**  **Supporting Information—Fig. S2****]**. Stems of *B. oldhamii* are usually green, but there were periods when mature stems turned yellow and the plants appeared to be dormant, with no positive pressure detected during these time periods. The yellowing was reversible, and stem pressure usually resumed after these periods of dormancy. Maximum pressures above 450 kPa were registered for 16 days of the one-year measuring period **[see**  **Supporting Information****]**, but pressure sensors were not calibrated above 450 kPa, so the true maximum pressure in this species is uncertain.

**Figure 4.**
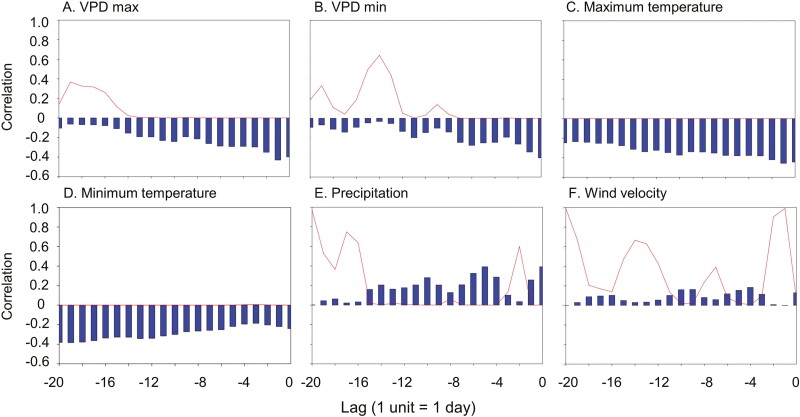
Cross correlations between daily maximum stem pressure measurements and daily weather data between July 2020 and July 2021. The x-axis represents the time lag of the correlation, with one unit representing one day. Time lag zero shows the correlation of stem pressure with the current weather, and negative lags show correlation of stem pressure with weather conditions over the previous 20 days. Bars indicate the correlation coefficients, and the line indicates the statistical probability of the correlation without correction for multiple comparisons.

Cross-correlation analyses between daily maximum stem pressure and weather data found a variety of significant relationships (Fig. 4). Stem pressure was negatively cross-correlated with the maximum (i.e. mid-day) and minimum (i.e. night-time) VPD of the current day and several days earlier, indicating that dry atmospheric conditions were followed by lower stem pressures (Fig. 4a, b). The hypothesis that the magnitude of nocturnal pressure would be positively correlated with VPD during midday was rejected, but the hypothesized negative cross-correlation between stem pressure and nighttime VPD was supported. Stem pressure was also negatively cross-correlated with maximum and minimum daily temperatures of the current and previous days (Fig. 4c, d), most likely because high temperatures in California are almost always associated with high VPD. Stem pressures were positively cross-correlated with precipitation of previous days (Fig. 4e) even though the plants were well-watered at all times, and there was no consistent cross-correlation with maximum wind velocity (Fig. 4f).

**Figure 5.**
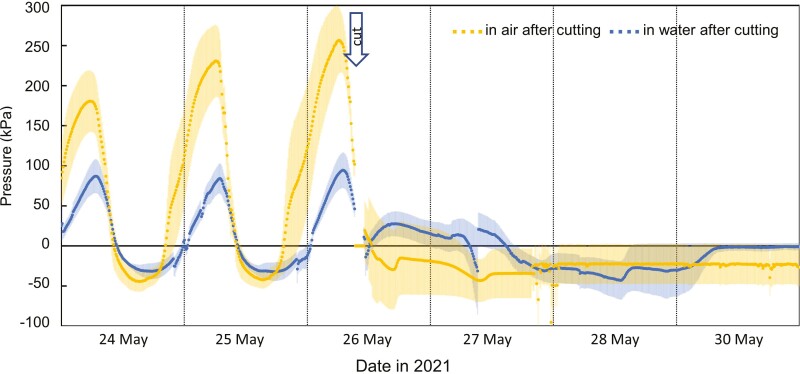
Effects of cutting stems of *B. oldhamii* on mean stem pressure in the cut pieces after submerging the lower end in water (mean of *n* = 4) or leaving it in air (mean of *n* = 4). Stems were cut around 9:15 am on 26 May 2021. The coloured shaded areas for each line show standard errors. After cutting, stems in water had positive pressure significantly above zero between 26 May from 15:11 to 18:41, as determined by one-sided 95% confidence intervals, while stems in air never showed pressures above zero.

Cutting experiment 1 was conducted to determine if cut stem segments with roots or rhizomes would continue to produce positive pressure when in contact with a water source. This was indeed the case (Fig. 5). Segments in contact with water continued to produce moderate positive pressure (as determined by 95% confidence intervals above 0 kPa) for several hours, while segments in air did not produce positive pressure. We observed that cutting caused rapid formation of tyloses in all cut vessels, rendering vessels non-functional and this may account for the rapid cessation of pressure in stems that were in touch with water. The random assignment of replicates to treatments accounts for stems assigned to the ‘no water’ treatment having higher positive pressures before cutting than the ‘water’ treatment. However, this difference reversed after cutting and the application of treatments (Fig. 5).

**Figure 6.**
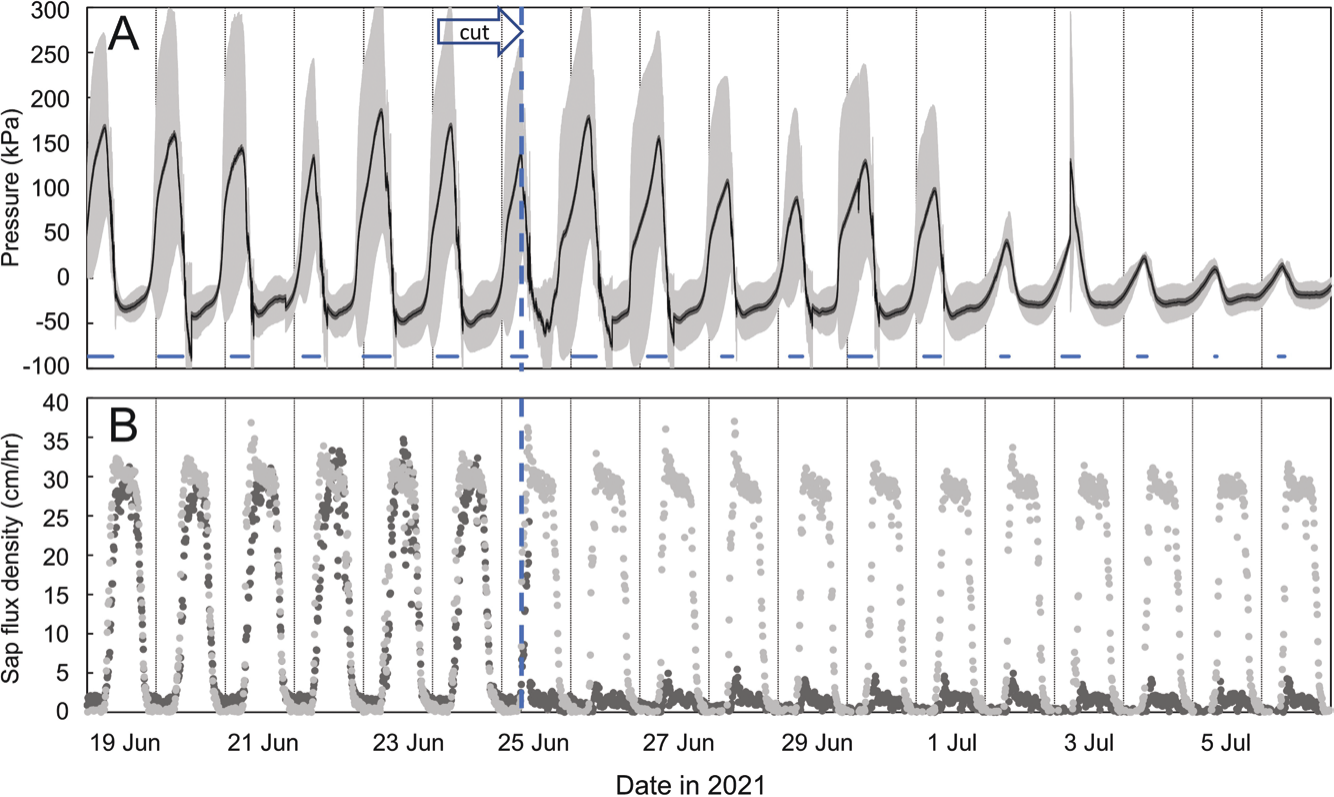
Effects of cutting the stem of *B. oldhamii* at 2.4 m above soil level on stem pressure and sap flow below the cut. The arrow indicates the time of cutting (11:00 on 25 June 2021). a. Mean stem pressure (mean of *n* = 3, ± SE) in the cut stem. The horizontal bars under the curve indicate times at which all three stems had positive pressures. b. Sap flux density in the cut stems (mean of *n* = 3) compared to an uncut control stem (*n* = 1) of the same plant.

We hypothesized that the buildup of positive pressure in the evening would be triggered by the cessation of transpiration-driven sap flow and that resumption of sap flow in the morning would cause the pressure to dissipate. The second cutting experiment, where stems were cut at 2.4 m above soil level, led to rejection of that hypothesis. Even though the cutting prevented sap flow, the diurnal pressure patterns continued as before in all three cut stems for at least 8 days (Fig. 6), showing that there was no causal link between sap flow and stem pressure. The onset of positive pressure in uncut and cut stems in this experiment varied from between midnight and 7:50 am, but the pressure consistently disappeared sometime between 8:07 and 9:08 am (Pacific Standard Time), both before and after cutting, which was about 3 hr after sunrise.

## Discussion

### Where is positive xylem pressure generated in bamboo?

Positive nocturnal stem pressure in the bamboo species studied is clearly not generated in roots (Figs. 1 and 5). This contradicts a universal assumption that there is ‘root pressure’ in bamboo (e.g. Cochard *et al.* 1994; Ewers *et al.* 1997; Marine 2009; Wang *et al.* 2011; Cao *et al.* 2012; Yang *et al.* 2012; Mei *et al.* 2016), which has never been tested before. Yang *et al.* (2024) even modelled root pressure in bamboo without ascertaining first if pressure in these plants is actually generated in roots. Only one species was included in our study, but it is unlikely that this species is the only bamboo that generates pressure in stems and rhizomes rather than in roots. That said, additional experiments with other bamboo species and genera are warranted, including to determine if there are differences between species with clumped vs. runner rhizome morphology and if large differences in stem anatomy between bamboos (Grosser and Liese 1971) are related to differences in pressure generation.

Moreover, pressure generation in bamboo also does not require a rhizome and can be generated in stems that are detached from roots and rhizomes (Fig. 5). The observation that pressures in rhizomes and basal internodes do not always exceed pressure measured higher up in the stem (Figs. 1–3) also indicates that stem pressures are generated locally in each internode and do not originate from the rhizome or solely from the base of the stem.

### How is positive xylem pressure generated in bamboo?

These findings beg the question as to how stem pressure is generated in bamboo. Our experiments and measurements do not fully answer that question, but we can narrow down the possibilities based on four distinct observations. (i) The pressure is not generated as osmotic pressure in stem vessels, because vessels do not contain semi-permeable membranes for solutes that would allow a build-up of osmotic pressure. Also, stem segments in touch with a water source continued to produce positive pressure for at least one day (Fig. 5). (ii) The pressure is not generated solely in rhizomes or basal stem internodes, because pressure can be generated without rhizomes (Fig. 5) and because pressure higher up can sometimes equal or exceed pressure near the base (Figs. 1–3). This means that the pressure is generated at least in part locally in each stem internode, most likely influencing pressure in adjacent internodes as well. (iii) The dissipation of pressure in the morning coincides with the resumption of transpiration and sap flow but is not caused by the resumption of sap flow. Stem pressure dissipated in the morning even in the absence of sap flow (Fig. 6). If sap flow is not causing the pressure to dissipate then the pressure clearly does not exist solely in vessels and must originate from other stem tissues. (iv). Following from the previous observation, the buildup and dissipation of nocturnal pressure must be caused either by metabolic processes with pronounced day-night patterns, possibly involving stem photosynthesis and/or by diurnal physical or hydraulic factors, such as nocturnal water uptake that leads passively to swelling of cells and tissues and could involve nocturnal expression of aquaporins (Sun *et al.* 2018).

Together, these observations suggest that stem pressure in bamboo must originate in the tissues of each rhizome and stem internode in which it is found or possibly in the nodes. Besides xylem, these bamboo tissues include phloem, living parenchyma and massive fibre bundles. The latter in the genus *Bambusa* include vascular fibre bundles that cap metaxylem vessels, protoxylem and phloem, as well as extravascular fibre bundles (Grosser and Liese 1971). Our stem diameter measurements show that stem pressure is directly associated with stem swelling, which is indicative of higher water content [Figs. 1 and 2 and **see** Supporting Information—Figs. **S1 and S2**]. Our findings do not allow us to conclude which stem tissues are involved in pressure generation. The phloem is an obvious option for the generation of nocturnal osmotic pressure that results in high turgor pressure (Schenk *et al.* 2021). Parenchyma cells can also swell and generate pressure, and bamboo fibres contain a relatively low percentage of lignin (Li *et al.* 2010; Yuan *et al.* 2021), so they could potentially swell as well. The idea of pressure generated by swelling tissues may bring to mind the tissue pressure hypothesis proposed by Canny (1995) as an alternative to the cohesion-tension theory. While Canny’s hypothetical mechanism cannot work for moving sap flow during the day (Comstock 1999; Stiller and Sperry 1999; Tyree 1999), such a mechanism could potentially work for creating stem pressure in the absence of sap flow during the night when pressure cannot be dissipated except by the small amounts of ‘bamboo rain’ (Molisch 1898) guttation from the leaf tips.

### The functional significance of positive xylem pressure

Bamboos generate positive nocturnal pressure when they have access to an abundance of soil water, including in our study when xylem embolism is unlikely to be a problem for these well-watered plants. The lowest stem water potential we observed was about −1 MPa in July 2020 **[see**  Supporting Information—**Fig. S2****]**, and it is unknown if that is low enough to induce embolism in this species. If it is then positive nocturnal pressure could potentially refill gas-filled vessels. However, positive nocturnal pressure in bamboo and most other plants that show guttation at night (Singh 2016*a*) occurs not only after drought periods, but whenever VPD is low (Fig. 4a, b) and soil water availability is high (Fig. 4e). It appears to be a phenomenon that normally occurs in the absence of current or previous drought stress. What then may be the physiological function of nocturnal stem pressure in bamboo? Many bamboo species grow in the understory of wet tropical forest, where transpiration may often be too low to transport minerals and hormones to the leaves efficiently. Feild and Arens (2007) hypothesized that understory plants from wet tropical environments may have evolved positive pressure as a way to move resources to the leaves, and this may be true for bamboos. Even though *B. oldhamii* is a tall bamboo species that is not restricted to forest understories, nocturnal pressure may still benefit the plants by rehydrating stem and leaf tissues overnight and allowing for high transpiration rates during the day, as found for other bamboo species (Saha *et al.* 2009; Yang *et al.* 2012, 2015).

### Conclusion and outlook

While more research is needed to elucidate the mechanisms behind positive nocturnal stem pressure in bamboo and more species must be studied, it is clear that the pressure does not originate in roots and is not ‘root pressure’. It is remarkable that none of the many previous studies regarding positive pressure in bamboo included a test to determine where the pressure originated. If the location of the pressure generation remains unknown then it is little wonder that the subject of positive pressure in plants has largely remained an unsolved mystery of plant biology (Singh 2016*b*; Schenk *et al.* 2021) ever since positive pressure measurements were first reported by Stephen Hales in his book ‘Vegetable Staticks’ (Hales 1727).

Our findings move the positive pressure mystery away from being considered an osmotic phenomenon in root vessels towards a metabolic or physical process in stems, at least for bamboo. It remains to be seen if a similar process occurs in other plants, including other grasses. Isolated roots of maize are capable of generating positive pressure (Steudle *et al.* 1987; Dustmamatov and Zholkevich 2008), so there is no doubt that root pressure occurs in that species. It is possible that in grasses stem-generated pressure is unique to the pseudo-woody bamboos. It is an open question if stem-generated pressure also exists in other plants from moist tropical environments, such as plants from the basal angiosperm lineages that frequently show leaf guttation (Feild and Arens 2007). Hopefully, deployment of pressure sensors on roots, rhizomes and stems of many more plant species will help to answer these questions.

## Supporting Information

The following additional information is available in the online version of this article –

Figure S1. Cross correlations between stem pressure and stem diameter fluctuations, between stem pressure and sap flow, and autocorrelations within the three variables.

Figure S2. Stem water potential of *Bambusa oldhamii*, measured with a single stem psychrometer (model PSY1, ICT International, Armidale, NSW, Australia) installed at 1 m stem height from 7 to 10 July 2020.

Table S1: Daily data for July 2020 to July 2021. Weather data are for the Fullerton Airport. Stem pressure, stem diameter fluctuations, and sap flow are for *Bambusa oldhamii*. These data were used for the cross-correlations shown in Figure 4 and include the time periods shown in Figures 1–3.

Table S2: Hourly data for stem pressure, sap flow, and stem diameter fluctuations of *Bambusa oldhamii* in July 2020. Parts of these data are shown in Figure 2. These data were used to calculate cross-correlations between the three variables shown in see Supporting Information—**Fig. S1A and C**, and autocorrelations in **Supporting Information—Fig. S1E**.

Table S3: Hourly data for stem pressure, sap flow, and stem diameter fluctuations of *Bambusa oldhamii* in March 2021. Parts of these data are shown in Figure 1. These data were used to calculate cross-correlations between the three variables shown in **Supporting Information—Fig. S1B and D**, and autocorrelations in **Supporting Information—Fig. S1F**.

plae040_suppl_Supplementary_Figures_S1-S2

plae040_suppl_Supplementary_Tables_S1-S3

## Data Availability

The data underlying this article are available in the article and in its Supporting Information. Raw data, including replicates, for all figures in this article will be shared on reasonable request to the corresponding author.
