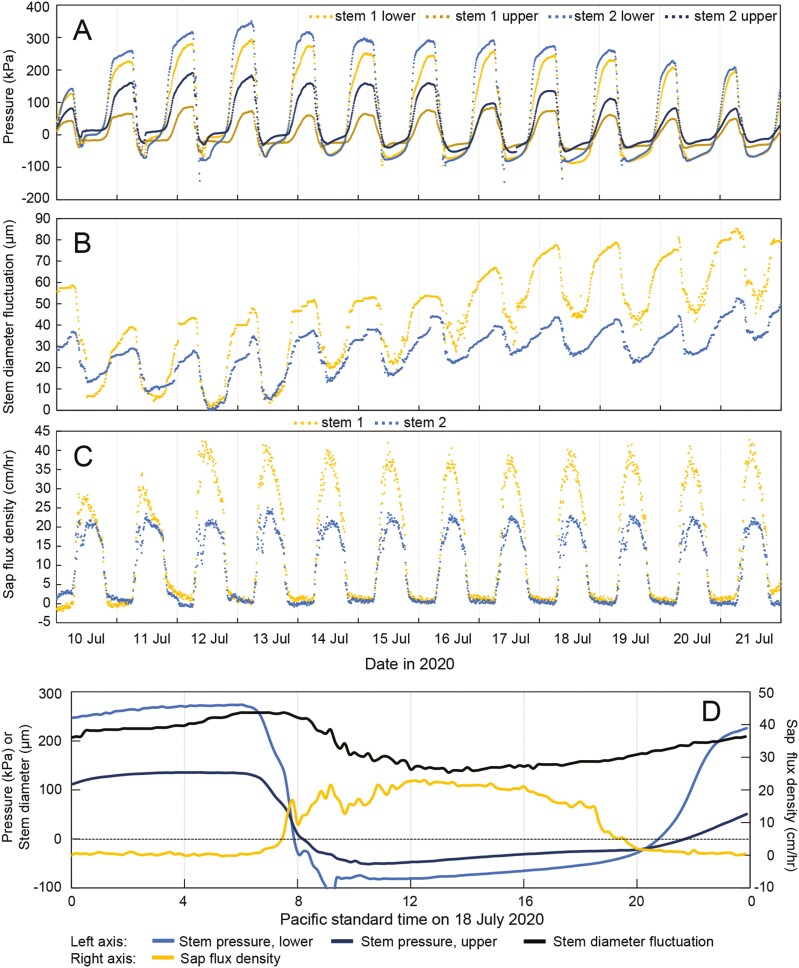# Correction to: Positive pressure in bamboo is generated in stems and rhizomes, not in roots

**DOI:** 10.1093/aobpla/plae057

**Published:** 2024-11-06

This is a correction to: Joseph M Michaud, Kerri Mocko, H Jochen Schenk, Positive pressure in bamboo is generated in stems and rhizomes, not in roots, AoB PLANTS, Volume 16, Issue 4, July 2024, plae040, https://doi.org/10.1093/aobpla/plae040

In the originally published version of this manuscript, the image of Figure 2 erroneously duplicated the image of Figure 1.

The manuscript has been amended to correct Figure 2.