# Supplementary material for: Positive pressure in bamboo is generated in stems and rhizomes, not in roots
Source: AoB Plants. 2024 Jul 19;16(4):plae040. doi: 10.1093/aobpla/plae040 (PMC11306578; doi:10.1093/aobpla/plae040)
Supplement: plae040_suppl_Supplementary_Figures_S1-S2 [file plae040_suppl_supplementary_figures_s1-s2.pdf]

Supplementary information

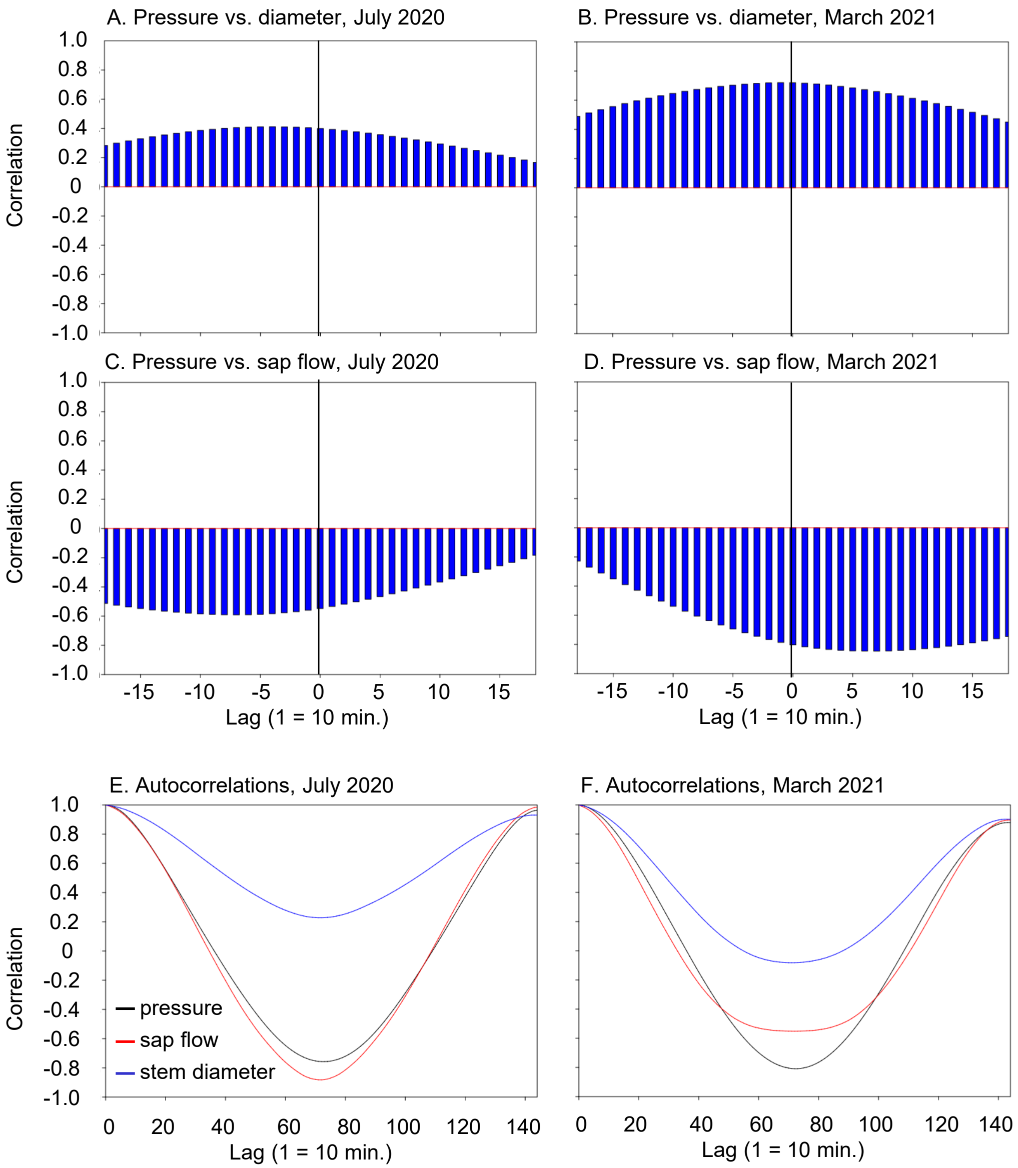

Figure S1. Cross correlations between stem pressure and stem diameter fluctuations for A. 10-21 July 2020 and B. 10-24 March 2021, as well as between stem pressure and sap flow for C. 10-21 July 2020 and D. 10-24 March 2021. One unit of lag on the x-axis represents a 10-minute measurement interval, with the x-axis showing cross correlations over ±180 minutes. A negative cross-correlation time lag represents stem pressure leading the other variable, while a positive time lag represents the other variable leading stem pressure. Blue bars in panels A-D indicate the correlation coefficients, and the red line indicates the statistical probability of the correlation, uncorrected for multiple comparisons. Autocorrelations within the three variables over 144 lags of 10 minutes each (one day) are shown for E. 10-21 July 2020 and F. 10-24 March 2021. Note that all three variables are highly autocorrelated at the time scale of three hours represented in panels A-D..

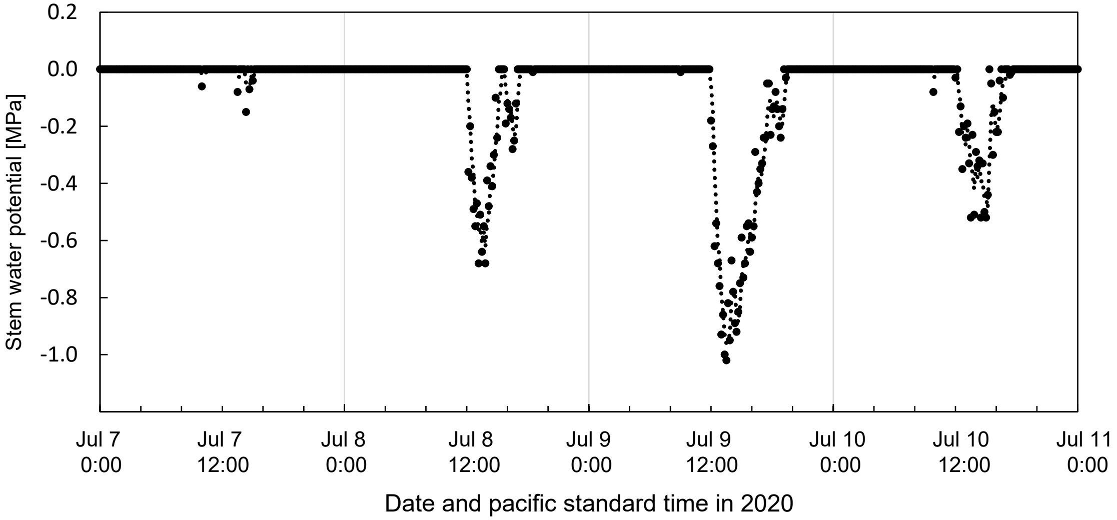

Figure S2. Stem water potential of *Bambusa oldhamii*, measured with a single stem psychrometer (model PSY1, ICT International, Armidale, NSW, Australia) installed at 1 m stem height from 7-10 July 2020 (on stem 1, the same as in Figure 2). The measurements show an artefact caused by nightly refilling of the psychrometer chamber with liquid water, caused by positive stem pressure. During this measurement period, the chamber appears to have emptied around noon, making the psychrometer briefly functional before the chamber apparently refilled in the evening. This was the only time when stem water potential measurements were briefly possible. Filling of the chambers made all other attempts unsuccessful.
